# Rapid response of locally advanced oral squamous cell carcinoma to apatinib: A case report

**DOI:** 10.1515/med-2021-0360

**Published:** 2021-11-03

**Authors:** Jian He, Yangyang Zhang, Jin Gao, Liting Qian

**Affiliations:** Department of Radiation Oncology, The First Affiliated Hospital of USTC, Division of Life Sciences and Medicine, University of Science and Technology of China, Hefei, Anhui Province, 230031, People’s Republic of China; Department of Radiation Oncology, Anhui Provincial Cancer Hospital, Hefei, Anhui Province, 230031, People’s Republic of China

**Keywords:** apatinib, oral cancer, head and neck squamous cell carcinoma, VEGFR-2

## Abstract

There are no effective therapeutic options for locally advanced head and neck squamous cell carcinoma (HNSCC). Additionally, there is no standard therapy for patients subjected to multiple lines of treatment. Angiogenesis plays a key role in tumor growth and metastasis. Therefore, inhibition of tumor angiogenesis is an important strategy for tumor therapy. Apatinib is a novel tyrosine kinase inhibitor that inhibits angiogenesis by targeting vascular endothelial growth factor receptor-2 (VEGFR-2). The effect of apatinib on HNSCC has not been clearly established. In this study, we administered apatinib in combination with anti-epidermal growth factor receptor (EGFR) targeted and systemic chemotherapy for the treatment of oral cancer and to achieve better disease outcomes. To avoid fatal bleeding, after achieving good clinical outcomes, the follow-up treatment plan was adjusted. The efficacy of apatinib combined with anti-EGFR targeted and systemic chemotherapy for the treatment of oral cancer has not been previously reported. Our findings show the therapeutic potential of apatinib for advanced HNSCC patients with multiple lines of chemotherapy, especially for patients with large neck masses.

## 1 Introduction

Oral cancer is a malignant tumor that occurs in the oral and maxillofacial regions, and approximately 90% is oral squamous cell carcinoma (SCC). It is the most common SCC of the head and neck. Annually, there are about 350,000 new cases and over 170,000 mortalities associated with oral cancer, globally accounting for 2.0 and 1.9% of new morbidity and mortality cases, respectively [1]. Patients with HNSCC in the early stages are mainly treated with surgery or radiotherapy, while patients with advanced stage HNSCC are treated with surgery, radiotherapy, and chemotherapy as the main therapeutic options. However, the overall efficacy is not good. The 5-year survival rate is only 40–50%, while 40–60% of patients have local recurrence or distant metastasis [2]. Therefore, there is a need to develop new therapeutic options for HNSCC. Apatinib mesylate is a small angiogenesis inhibitor that targets VEGFR-2, which in turn inhibits tumor angiogenesis and tumor growth [3]. Clinically, apatinib, which has a favorable safety profile and efficacy, has been used for the treatment of some advanced cancers. However, the efficacy of apatinib in HNSCC is unknown. In this study, we report the clinical outcomes of a patient with locally advanced HNSCC after apatinib combined with anti-EGFR targeted and chemotherapy. Our findings show that apatinib has a favorable efficacy for the treatment of HNSCC.

## 2 Case report

**Figure 1:**
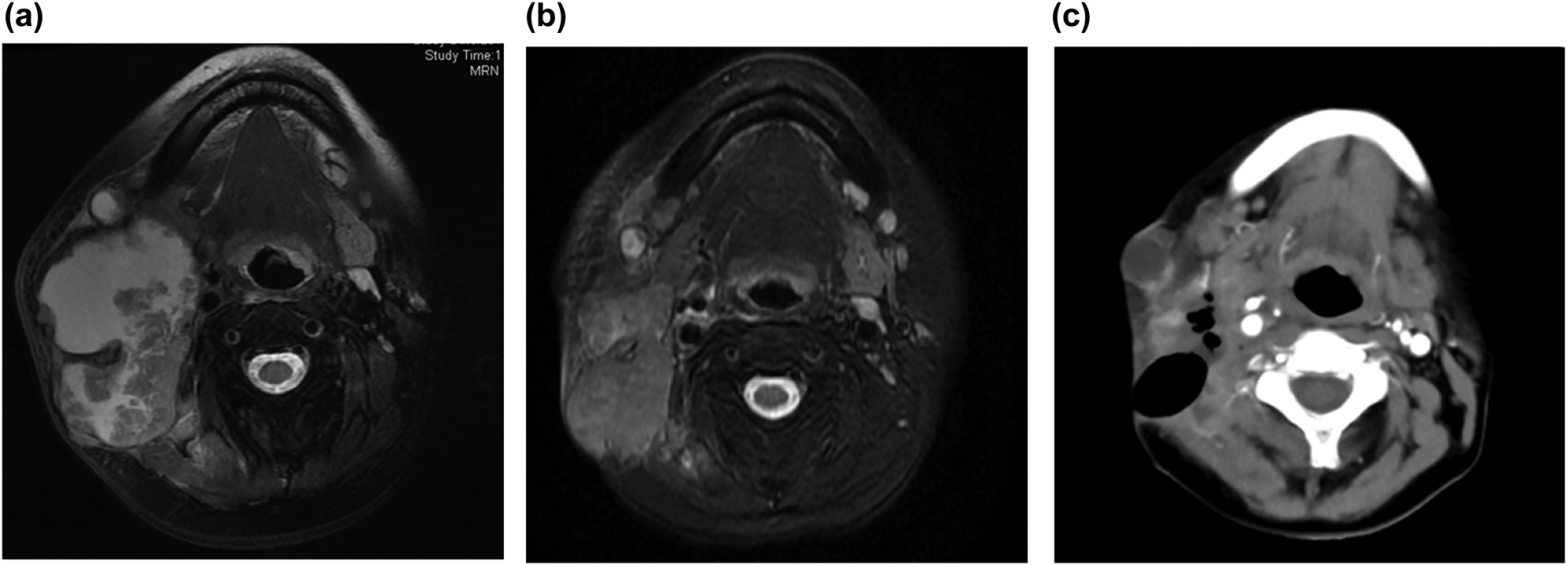
Images of neck tumor. (a) Before second-line chemotherapy, MRI revealed a soft tissue mass with a volume of about 7.5 cm^3^ × 5.5 cm^3^ × 7 cm^3^ on the right neck that invaded adjacent muscles and skin, and multiple enlarged cervical lymph nodes. (b) After the second cycle of second-line chemotherapy, MRI examination revealed a slight decrease in the volume of the tumor. (c) After apatinib treatment for 10 days, the computed tomography revealed the neck tumor was markedly reduced.

**Figure 2:**
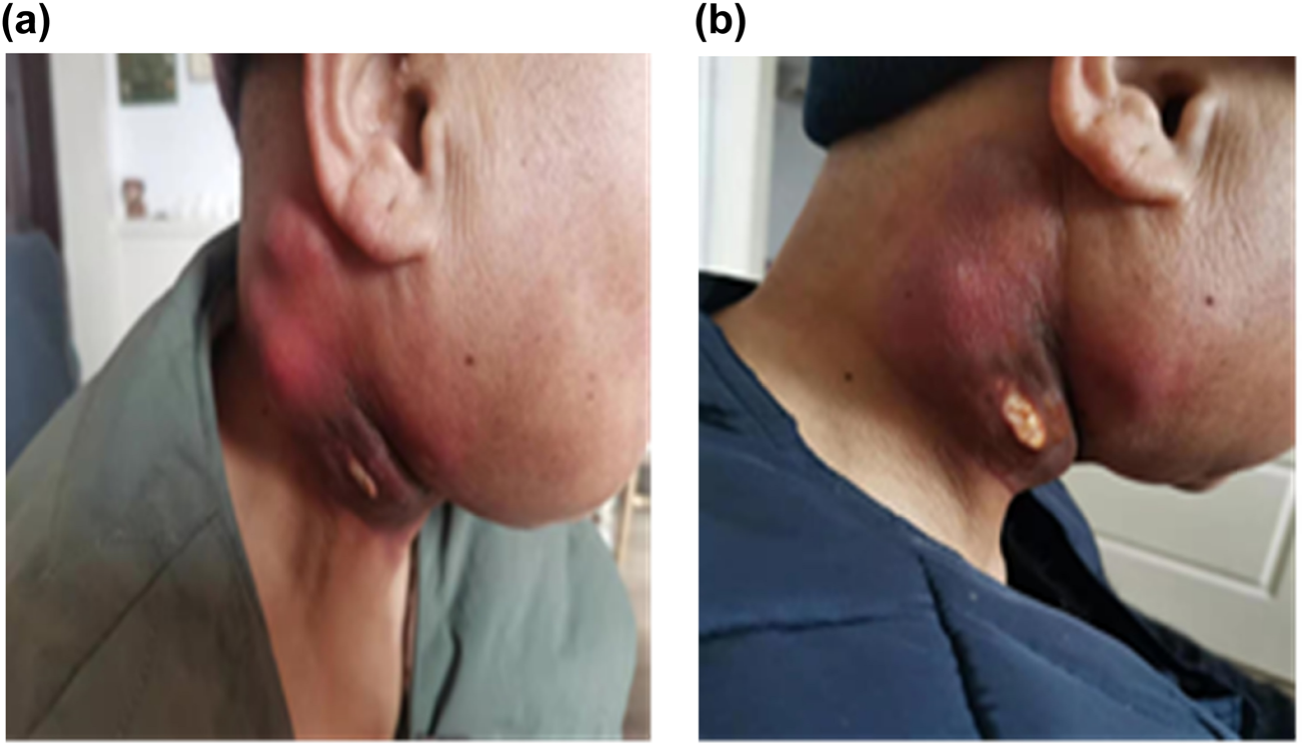
Clinical pictures of neck tumor. (a) Before treatment with apatinib on 16 December 2019. (b) After apatinib treatment for 5 days on 21 December 2019.


A 56-year-old woman presented with a 3-month history of a painful oral ulcer and local mass in the right cheek. The patient denied any history of hypertension, diabetes, coronary heart disease, smoking, drinking, or hereditary disorders. Physical examination revealed a mass of about 8 cm^3^ × 6 cm^3^ × 7.5 cm^3^ on the right neck that invaded adjacent muscles and skin, with an irregular shape, unclear boundaries, and poor movement. There was an ulcer of about 1.5 cm^2^ × 2 cm^2^ in the buccal mucosa. Her routine blood and blood biochemical parameters were normal. Her serum SCC antigen level was 2.4 ng/mL (reference range: <1.5 ng/mL), while carcinoembryonic antigen (CEA) level was within normal ranges. The pathological biopsy (right buccal mucosa), which was performed in the dental outpatient clinic, revealed highly differentiated SCC. Immunohistochemistry staining indicated VEGF (+). B-ultrasound revealed a solid cystic mass in the right submandibular with multiple lymphadenopathy. The positron emission tomography computed tomography (PET-CT) scan revealed a deep cystic solid mass in the posterior margin of the right parotid gland with uneven fluorodeoxyglucose uptake. The mass involving surrounding tissues, multiple metastatic cervical lymph nodes, with no clear boundary with the right common carotid artery and no metastasis in other parts. The diagnosis was oral SCC at clinical stage T2N3bM0 (IVB). The patient’s body surface area (BSA) was 1.53 m^2^. The first-line chemotherapeutic option was intravenous paclitaxel (135 mg/m^2^) on day 1 and cisplatin (30 mg/m^2^) on day 1–3. However, there was no decrease in neck mass volume after one cycle. Magnetic resonance imaging (MRI) revealed a soft tissue mass with a volume of about 7.5 cm^3^ × 5.5 cm^3^ × 7 cm^3^ on the right neck that invaded adjacent muscles and skin and multiple enlarged cervical lymph nodes (Figure 1a; Table 1). According to the Response Evaluation Criteria in Solid Tumors (RECIST) version 1.1 standard, the tumor was considered as stable disease (SD). Then, the treatment was adjusted to nimotuzumab (200 mg) combined with paclitaxel and cisplatin as second-line chemotherapeutic options, every 3 weeks. After one cycle, the mass became soft and slightly decreased. However, the mass did not continue to shrink after the second cycle of second-line chemotherapy. MRI examination revealed a slight decrease in the volume of the tumor compared to pre-second-line chemotherapy (Figure 1b; Table 1). After consultation with an oncologist and head and neck surgeon, given the large neck mass, insensitive treatment, cost of treatment, and VEGF (+), apatinib was administered (500 mg/day) on 16 December 2019 (Figure 2a). After only 5 days of treatment, the size of the neck mass had significantly reduced and was almost flat (Figure 2b). After apatinib treatment for 10 days, the tumor continuously shrunk. There were no occurrences of hypertension, hand-foot syndrome, proteinuria, oral ulcer, or hematologic toxicities. However, due to rapid tumor shrinkage, a local skin ulceration with exudate was formed (Figure 1c; Table 1). Subsequently, the wound was actively treated with oral cefuroxime sodium, local cleansing, and dressing changes. After a discussion with multiple disciplinary teams, apatinib was withdrawn on 23 January 2020, and the treatment was changed to low-dose maintenance chemotherapy because the patient declined radiotherapy. The specific plan was paclitaxel plus capecitabine every week. Unfortunately, the patient died of a pulmonary infection 3 months later.

**Table 1:** The volumes of the tumor at different time

Time	Before second-line chemotherapy	After second-line chemotherapy	After apatinib treatment for 10 days
Tumor volume (cm^3^)	7.5 × 5.5 × 7.0 (288.75)	5.3 × 3.4 × 7.0 (126.14)	1.5 × 1.9 × 2.0 (5.70)

**Ethics and consent statements:** This study was approved by the ethics committee of Anhui Provincial Cancer Hospital, and written informed consent was obtained from the patient.

## 3 Discussion

Due to the atypical symptoms and signs of oral cancer, more than 60% of patients are diagnosed at stage III or IV. They lose the chance for surgery and can only be comprehensively treated by radiotherapy and chemotherapy [4]. Neoadjuvant chemotherapy can enhance the local control rate of head and neck tumors or the rate of distant metastasis. For patients with unresectable, recurrent, and metastatic head and neck tumors, paclitaxel plus platinum drugs are the recommended chemotherapeutic options [5]. The patient’s tumor was large, and boundaries with internal jugular arteriovenous and peripheral muscles were unclear, implying that the patient could not be operated. Paclitaxel plus cisplatin were administered as first-line chemotherapeutic options. However, after one cycle, there was no obvious decrease in the volume of neck mass. EGFR is closely associated with tumor cell proliferation and metastasis. EGFR is highly expressed in most HNSCC, thereby affecting the prognosis of HNSCC [6]. Anti-EGFR targeted combination therapy can significantly prolong the progression-free survival (PFS) and overall survival (OS) of patients with advanced HNSCC. Moreover, it can significantly improve the patient’s quality of life [7,8]. Clinically applied anti-EGFR monoclonal antibodies include cetuximab and nimotuzumab. However, due to the high cost of anti-EGFR monoclonal antibodies, the patient preferred the combinational therapy of nimotuzumab, paclitaxel, and cisplatin. After two cycles, the mass became soft and slightly decreased.

Tumor angiogenesis plays a key role in tumor growth and metastasis. For continuous cell proliferation, tumor tissues rely on oxygen and nutrients provided by neovascularization. The VEGF/VEGFR signaling pathway is considered the most critical step in tumor angiogenesis. VEGFR inhibitors can bind VEGF, which suppresses the binding of VEGF to VEGFR on the surface of vascular endothelial cells, thereby inhibiting biological effects, including tumor micro angiogenesis, invasion, and metastasis [9]. Apatinib was approved by China Food and Drug Administration (CFDA) in 2014 for the treatment of late-stage gastric adenocarcinoma or gastric esophageal junction adenocarcinoma that has progressed or relapsed after treatment with at least two lines of systemic chemotherapy.

Apatinib has been clinically used for various late-stage cancers, including hepatocellular carcinoma [10], non-small cell lung cancer [11], osteogenic sarcoma [12], colorectal cancer [13], and cervical cancer [14], with a favorable safety and good efficacy. However, the efficacy of apatinib in HNSCC has seldom been reported, with only individual case reports. Meng et al. [15] reported that the combination of apatinib and S-1 in the treatment of three cases of advanced head and neck tumors achieved partial responses and mild adverse reactions. The efficacy of apatinib combined with anti-EGFR targeted and systemic chemotherapy for the treatment of oral cancer has not been previously reported. In this study, we administered apatinib in combination with nimotuzumab and chemotherapy. The neck tumor was significantly reduced after treatment with apatinib for only 5 days. Since the patient had a large neck mass that was rich in blood vessels, apatinib rapidly exerted antiangiogenic effects. Therefore, the tumor tissue could not be supplied with enough oxygen and nutrients for continuous proliferation, thereby causing tumor necrosis and shrinkage. Zhu et al. [16] reported that apatinib enhances the risk of fatal hemorrhage. This is because during growth, the tumor invades blood vessels, and after apatinib administration, the tumor shrinks, thereby causing bleeding. In addition, drug-induced coagulation dysfunction and reduction of vascular endothelial growth factor synthesis destroy vascular integrity, which may increase the risk of bleeding [17]. To avoid fatal bleeding, we adjusted the follow-up treatment plan in time and achieved good outcomes.

Common side effects of apatinib, including hypertension, oral ulcer, hand-foot syndrome, proteinuria, and fatigue, are generally manageable and acceptable. The patient did not present these side effects, which were attributed to the short-term application of apatinib. The possible reasons for pulmonary infection in this patient were: i. Poor immunity due to the effects of cancer, making her susceptible to lung infections; ii. Pulmonary metastases, pleural effusions, and other coinfections in the lungs; and iii. Bone marrow suppression and immune deficiency after chemotherapy, which led to pulmonary infections. A direct association between pulmonary infection and apatinib has not been reported. This patient was administered with oral apatinib for one month and did not present any symptoms of pulmonary infection during this period; therefore, it was very unlikely that apatinib caused pulmonary infection. Studies should determine whether apatinib can cause pulmonary infections.

In this case, apatinib played a rapid and overwhelming role in tumor control. However, for clinical applications of apatinib, various considerations should be paid attention to. First, apatinib is effective for advanced HNSCC patients with multiple lines of chemotherapy treatment, especially in patients with large neck masses. Apatinib can be used in combination with anti-EGFR targeted and systemic chemotherapy, as described in our case. However, the role of apatinib in HNSCC should be confirmed in large-scale prospective clinical trials. Second, there is a need to establish the appropriate cohort of patients to receive apatinib. HNSCC patients with tumor invasions of large blood vessels have a high risk of fatal bleeding; therefore, apatinib should be used with caution. Third, tumor regression and necrosis are closely observed during the treatment of apatinib. Meanwhile, the coagulation function should be regularly monitored, and the treatment plans should be adjusted in time to avoid the risk of fatal bleeding.

In conclusion, apatinib combined with anti-EGFR targeted therapies and chemotherapy may be effective for advanced HNSCC patients, especially for patients with large neck masses to avoid the occurrence of fatal bleeding. However, the efficacy and safety for apatinib should be confirmed by multicenter large-scale prospective clinical trials, which may provide an effective treatment modality for HNSCC patients.

## Abbreviations

HNSCC: head and neck squamous cell carcinoma
VEGFR-2: vascular endothelial growth factor receptor-2
EGFR: epidermal growth factor receptor
SCC: squamous cell carcinoma
CEA: carcinoembryonic antigen
PET-CT: positron emission tomography computed tomography
BSA: body surface area
MRI: magnetic resonance imaging
RECIST: response evaluation criteria in solid tumors
SD: stable disease
PFS: progression-free survival
OS: overall survival
CFDA: China Food and Drug Administration
